# Does green stem photosynthesis affect plant drought tolerance and recovery in avocado?

**DOI:** 10.1093/aobpla/plaf044

**Published:** 2025-08-23

**Authors:** Nadia A Valverdi, Paula Guzmán-Delgado, Gregory R Goldsmith, Eleinis Ávila-Lovera

**Affiliations:** Schmid College of Science and Technology, Chapman University, 1 University Drive, Orange, CA 92866, United States; Department of Plant Sciences, University of California Davis, One Shields Ave., Davis, CA 95616, United States; Schmid College of Science and Technology, Chapman University, 1 University Drive, Orange, CA 92866, United States; Schmid College of Science and Technology, Chapman University, 1 University Drive, Orange, CA 92866, United States; Section (and Section Editor): Form & Function

**Keywords:** carbohydrates, hydraulic conductivity, *Persea americana*, plant water relations, stem recycling photosynthesis

## Abstract

Woody plants with green stems may have advantages over non-green-stemmed plants in that extra photosynthetic carbon gain has the potential to improve plant drought tolerance and aid drought recovery. However, most studies relating to green stem photosynthesis and drought tolerance have been conducted on non-horticultural plants under natural growing conditions. We investigated whether avocado green stem photosynthesis enhances drought tolerance and recovery. We applied light exclusion and drought treatments to 3-year-old potted trees of cultivars ‘Hass’ and ‘Fuerte’. Measurements of soil moisture, midday stem water potential, stem photosynthesis, bark chlorophyll concentration, concentration of sugars + starch and stem hydraulic conductivity were conducted before, during, and 3 weeks after rewatering. Green stems of avocado re-assimilate CO_2_, but values did not significantly differ between cultivars. We also found that light exclusion reduced stem photosynthesis by 65% in ‘Fuerte’ and 30% in ‘Hass’ although bark chlorophyll concentration was unchanged. Drought reduced stem photosynthesis by 60%. Following drought recovery, there were neither treatment nor cultivar effects on stem photosynthesis. We also observed no effect of light treatment on hydraulic conductivity, such that there is no clear effect of stem photosynthesis on drought tolerance of these avocado trees. However, we observed an increase in hydraulic conductivity during the drought period with an increase in the concentration of sugars in the sapwood and a decrease in the concentration of starch, suggesting osmotic adjustment. Nonetheless, the contribution of carbon gain through stem photosynthesis may not play a significant role in hydraulic functioning of avocado under these conditions.

## Teaser

Many tree species are capable of using both their leaves and their stems to produce sugars via photosynthesis. Sugars produced in the green stems of plants may play an important role in helping those plants avoid and recover from drought stress. This study applied drought and shading treatments to two different cultivars of avocado and measured the production of sugars in the bark and stem wood. Although both Hass and Fuerte stems do stem photosynthesis, we found no significant relationship between photosynthetic rates and the flow of water through those stems following drought, although there was evidence for a change in the concentration of sugars in the stems. Studying the mechanisms by which agriculturally important species such as avocado withstand drought is critical given projected changes in climate.

## Introduction

Drought stress is a significant threat to plant survival both in the wild (Zia et al. 2021) and for agricultural production (Toulotte et al. 2022). Hence, understanding the mechanisms behind plant drought tolerance is necessary to mitigate the negative effects of drought on plant survival, growth, and productivity. Among the traits known to mediate survival (Pivovaroff et al. 2016, Santiago et al. 2016), stem photosynthesis in woody plants has gained attention in recent years as it is widespread in seasonally dry ecosystems (Gibson 1983, Nilsen 1995), and it occurs at greater water-use efficiency than leaf photosynthesis (Ehleringer et al. 1987, Nilsen and Sharifi 1997), which can be important for plants facing water limitation.

Stem photosynthesis can be categorized as stem net photosynthesis or stem recycling photosynthesis (Ávila et al. 2014), with both types positively contributing to the carbon economy of plants either through assimilation of atmospheric CO_2_ or re-assimilation of previously respired CO_2_, respectively. In stem net photosynthesis, stems have stomata and/or lenticels and are able to assimilate atmospheric CO_2_, which produces a positive net CO_2_ assimilation rate when measured with an infrared gas analyser (IRGA). In stem recycling photosynthesis, stems are covered by a periderm with or without lenticels, and CO_2_ previously respired by inner tissues is re-assimilated. This process is evidenced by a decrease in the CO_2_ efflux in light compared with darkness. Stem photosynthesis challenges our notion that plants’ main carbon source is leaves and raises questions about the functional implications and ecological significance of stem photosynthesis in the context of water limitation.

**Figure 1.**
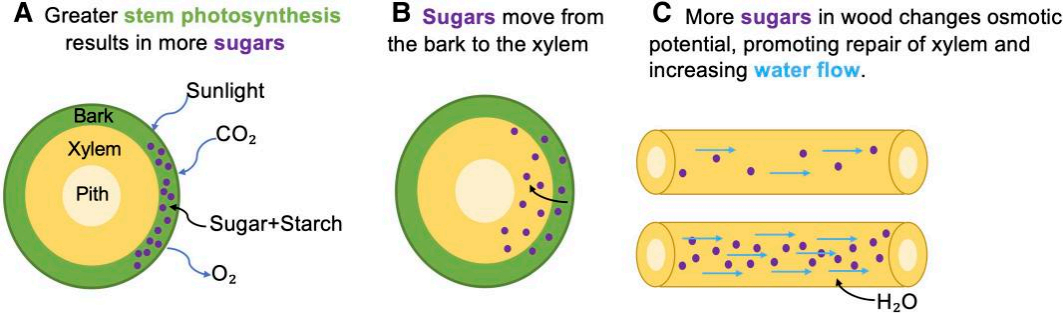
A conceptual figure demonstrating the relationship between stem photosynthesis and stem hydraulic conductivity. Figure by authors.

Recent research has suggested that stem photosynthesis may play an unexpectedly important role in the maintenance of stem water flow (i.e. hydraulic functioning) by facilitating the refilling of embolized xylem conduits with water by means of an osmotic gradient created by the presence of sugars that originate from stem photosynthesis (Fig. 1; Schmitz et al. 2012, Bloemen et al. 2016, De Baerdemaeker et al. 2017, Liu et al. 2019, Trifilò et al. 2021, Ávila-Lovera et al. 2024). Given the critical need to conserve water used for irrigated agriculture and ensure sufficient supplies to meet growing demands, it is important to understand the possible links between stem photosynthesis and drought tolerance in agriculturally important plants.

Previous studies on stem photosynthesis and its link to drought tolerance and recovery have been made mainly on native species in non-cultivated areas (De Roo et al. 2020b, Ávila-Lovera et al. 2024). Avocado (*Persea americana* Mill., Lauraceae) is an economically important crop, with a global production of 6 048 508 tons from 607 542 ha across 69 countries in 2017 (FAO 2019). In the USA, California produces ∼90% of the country’s avocado crop, with an annual market value of $383 million (California Avocado Commission 2019). Even though there are seven commercially grown cultivars in California, ‘Hass’ accounts for ∼95% of the total crop each year. However, recent drought events have threatened production because of the reduced water availability for irrigation (Pathak et al. 2018), which is of critical importance given avocado’s high sensitivity to drought compared with other specialty crop trees (Garrido and Vergara 2022). Therefore, the maintenance of physiological activity with limited water and/or capacity to recover after drought are important factors determining the resilience of avocado orchards. Both the saplings and terminal branches of mature avocado trees have green stems and recent measurements confirm that they are capable of stem photosynthesis (Valverdi et al. 2023b).

Could green stem photosynthesis be a mechanism contributing to avocado drought tolerance? And can photosynthetic carbon gain through stem photosynthesis help in the recovery of avocado trees by refilling xylem after drought?

We tested whether precluding stem photosynthesis by light exclusion had any effects on the production of non-structural carbohydrates (NSCs) and, in turn, led to changes in plant hydraulic function (e.g. stem conductivity) before, during, and after drought. We hypothesized that prolonged light exclusion would have both immediate effects on stem photosynthesis and non-NSCs and eventually on chlorophyll concentrations. In turn, we expected NSC concentrations in the stem bark would impact stem hydraulic conductivity by generating an osmotic gradient that would drive water back into impaired xylem (Salleo et al. 2004, Brodersen et al. 2010, Nardini et al. 2011). We also hypothesized that plants not subject to stem light exclusion would recover hydraulic function to levels before drought stress.

## Materials and methods

### Experimental design

We used 3-year-old potted trees of ‘Hass’ and ‘Fuerte’ avocado cultivars grafted on Zutano rootstock bought from Louie’s Nursery, Riverside, CA, USA. Trees were grown at the South Coast Research and Extension Center in Irvine, CA, USA in 20 l pots filled with potting media from the nursery with a trunk-cross-sectional-area average of 22.2 ± 4.6 cm^2^ for ‘Fuerte’ trees and 22.6 ± 3.9 cm^2^ for ‘Hass’ trees. Individual pots were arranged in a complete randomized block design with four individuals per cultivar (two cultivars) per block (four blocks) per level of light exclusion and water stress (two levels each; Supplementary Fig. S1). This corresponds to a total of 24 trees per block in a factorial design of 2 cultivars × 2 light treatments × 2 water treatments (*n* = 96 total trees). The trees were irrigated twice per day for 20 min, once at 08:00 and again at 17:00, from April through October of 2021. During wintertime, trees were irrigated once per day for 20 min at 08:00. The extension centre uses water reclaimed by the water district. Trees were fertilized with Triple Pro 15-15-15 (J.R. Simplot Company, Boise, ID, USA) in the month of February, and with Tropicote 15.5-0-0 calcium nitrate (YaraLiva, Yara, Tampa, FL, USA) in the months of June and September following nursery recommendations.

We applied stem light exclusion and water stress (drought) treatments, each of them with two levels (‘Control’ and ‘Painted’ and ‘Control’ and ‘Droughted’, respectively). First, the light exclusion was achieved by painting all the stems of the trees (main and laterals) using brown mineral-based paint (White Wash Plant Guard, IV Organic, Los Angeles, CA, USA) as paint has been found to be the most time-effective method to exclude light from stems in ecophysiological studies while also minimizing changes in stem temperature and relative humidity (Valverdi et al. 2023a). Trees were painted during the first week of July 2021 and were let to acclimate for 4 weeks before the first set of measurements.

Plants in the control treatment were maintained at a soil moisture of ∼35% (v/v) based on observations of pot field capacity (i.e. soil moisture following complete drainage from the bottom of the pot). Soil moisture was monitored towards the centre of each pot using a capacitive sensor with 12 cm rods (HydroSense II, Campbell Scientific, Logan, UT, USA) and averaged among cultivars and treatments. After the first measurements of stem photosynthesis, hydraulic conductivity, stem traits (e.g. chlorophyll concentration), and NSC concentration in bark and sapwood, drought was induced by stopping irrigation until soil moisture was reduced to ∼10% (v/v), stem water potential was close to −2 MPa in water-stressed plants, and leaves were observed to wilt and show mild necrosis (Supplementary Fig. S2). Here, our goal was to impose a drought that would approximate the level at which an orchard manager would turn on irrigation, even if the plants may be able to withstand additional drought. The severity of the stress imposed on the plants, as measured by stem water potentials, is in excess of that necessary to cause leaf embolism and necrosis (Cardoso et al. 2020, Garrido and Vergara 2022), inhibit stem growth (Celedón et al. 2012), and cause damage to fruits (Kaneko et al. 2024) in avocado trees of the same cultivars. Although we were unable to directly measure per cent loss of conductivity due to the destructive nature of those measurements, we monitored tree water status via midday stem water potential twice per week as described below. After ∼3 weeks, droughted trees were re-watered to restore the same moisture as controlled trees.

Measurements of stem photosynthesis, hydraulic conductivity, stem traits (e.g. chlorophyll concentration), and NSC concentration in bark and sapwood were completed three times across the experiment, *Before*, *During*, and *Post* application of the water stress treatment (Table 1). Since destructive sampling was done at the branch level, we sampled a different branch on the same tree at each sampling time (*Before*, *During*, and *Post*). The *Before* measurements were done 4 weeks after the trees were painted to analyse the effect of the light exclusion treatment alone. First, gas exchange measurements were performed, and then the following day, the same branch was collected for measurements of hydraulic conductivity, stem traits, and NSC concentration, as explained below. This procedure was repeated at all sampling times. The *During* measurements were completed 2 weeks after stopping irrigation (∼6 weeks after the start of the experiment). Irrigation was then re-started, and the *Post* measurements completed ∼3 weeks later.

**Table 1.** Description of measurements performed *Before*, *During*, and *Post* water stress induced in branches of potted ‘Hass’ and ‘Fuerte’ avocado trees.

Gas exchange	Functional traits	Hydraulic conductivity and architecture	Non-structural carbohydrates	Plant and soil water status
Stem photosynthetic re-assimilation rate (*A*_stem_, μmol m^−2^ s^−1^)	Bark chlorophyll concentration (Chlorophyll_a+b_, μg cm^−2^)	Stem hydraulic conductivity (*K*_h_, mg mm MPa^−1^ s^−1^)	Sugar concentration in the wood (mg g^−1^)	Midday stem water potential (ψ_stem-midday_, MPa)
		Sapwood-specific hydraulic conductivity (*K*_S_, mg mm^−1^ MPa^−1^ s^−1^)	Starch concentration in the wood (mg g^−1^)	Volumetric soil moisture concentration (%)
		Leaf-specific hydraulic conductivity (*K*_L_, mg mm^−1^ MPa^−1^ s^−1^)	Sugar concentration in the bark (mg g^−1^)	
			Starch concentration in the bark (mg g^−1^)	

#### Gas exchange

Gas exchange was measured in a terminal branch with a diameter of ≤0.5 cm between 10:00 and 14:00 using an IRGA (Li-6800, Li-Cor Biosciences, Lincoln, NE, USA) with a 3 × 3 cm leaf chamber hermetically sealed by lining the gaskets with terostat adhesive as per Ávila-Lovera et al. (2017). Measurements were conducted at 410 µmol mol^−1^ of CO_2_, 1500 µmol m^−2^ s^−1^ of photosynthetic photon flux density, 400 µmol s^−1^ of flow rate, 10 000 RPM of fan speed, 50% relative humidity, and 25°C of air temperature. Stem gas exchange was recalculated using total (not projected) stem surface area. Subsequently, stem photosynthetic rate (*A*_stem_), which turned out to be re-assimilation, was calculated using CO_2_ exchange values under light (*R*_light_) and dark (*R*_dark_) conditions as *A*_stem_ = |*R*_dark_ − *R*_light_| (Cernusak and Marshall 2000).

#### Plant water status

Midday stem water potential (ψ_stem-midday_) was measured with a Scholander pressure chamber (model 1000, PMS Instruments, Albany, OR, USA) between 12:00 and 14:00 twice per week throughout the experiment and on the same day and branch that gas exchange measurements were performed. Two leaves per branch were wrapped in aluminium foil and placed inside a plastic bag for at least 30 min to halt transpiration and ensure leaf water potential equilibrium with stem water potential before collection and measurement.

#### Stem hydraulic conductivity and hydraulic architecture

Native stem hydraulic conductivity (*K*_h_) was determined using the gravimetric method as described by Jacobsen (2014). The branch (≥60 cm in length) used for gas exchange and water potential measurements was collected the following day between 9:00 and 11:00 and the cut end was promptly sealed with parafilm. Branches were then enclosed in a black plastic bag and transported to the laboratory for immediate *K*_h_ measurements. Briefly, 20 mM KCl degassed solution was delivered from an elevated source into a stem segment, which was previously re-cut under the degassed solution (14 cm in length), through tubing and then into a reservoir placed on a balance. Estimation of *K*_h_ was based on the flow rate recorded by a computer connected to the balance divided by the pressure gradient driving the flow (pressure head divided by the length of the stem segment). Native sapwood- (*K*_S_) and leaf-specific (*K*_L_) hydraulic conductivity were calculated by dividing *K*_h_ by sapwood area (SA) and leaf area (LA), respectively. The SA was calculated by measuring the xylem and pith diameter at the basal end of the stem and subtracting the pith area from the area obtained using the xylem diameter. LA was determined for the leaves distal to the stem portion used for *K*_h_ using a digital scanner and ImageJ.

#### Bark total chlorophyll concentration

Bark total chlorophyll concentration (Chlorophyll_a+b_) was measured in bark segments as per Bruinsma (1963). The bark was carefully removed from a stem segment of known diameter and 2 cm in length, cut into small pieces and immersed in 2 ml of 80% acetone in water (v/v). The mixture was centrifuged at 1200 RPM for 5 min, and the supernatant was collected and diluted at a 1/1 ratio with 80% acetone (v/v). Absorbance was then measured at 645, 652, and 663 nm using a spectrophotometer (Cary 60 UV-Vis, Agilent Technologies, Santa Clara, CA, USA). The total chlorophyll concentration in the bark was calculated on a per-area basis using stem diameter and length.

#### Non-structural carbohydrate concentrations

Ten cm-long segments of the same stems used for measuring hydraulic conductivity per cultivar and treatment were used to evaluate NSC concentrations as per Zwieniecki et al. (2022). The stem sapwood and the bark were separated, dried at 75°C for 3 days, and ground into a fine (∼1 µm), homogeneous powder. For soluble carbohydrate or sugar extraction, 1 ml 0.2 M sodium acetate buffer, pH 5.5, was added to 25 mg of sample. The mixture was incubated at 70°C for 15 min, centrifuged at 15 000*g* for 10 min, and the supernatant diluted in bi-distilled water (1/20, v/v). Soluble carbohydrates were then quantified by adding 0.1% anthrone reagent dissolved in 98% sulphuric acid (m/v), incubating for 20 min at 100°C, cooling for 10 min, and reading absorbance at 620 nm in a spectrophotometer (Multiskan GO, Thermo Scientific). The remaining buffer and pellet were used for starch extraction. Samples were incubated at 100°C for 10 min to allow starch gelatinization and then cooled for 20 min. Then, 0.7 U of amylase and 7 U of amyloglucosidase were added, and samples were stirred in a rotary incubator at 37°C for 4 h to digest starch. Samples were subsequently centrifuged at 15 000*g* for 10 min, and the supernatant diluted in distilled water. Starch concentration was determined by subtracting pre-starch digestion soluble carbohydrate concentration from post-starch digestion soluble carbohydrate concentration. Two repetitions were performed per sample and the results were averaged.

### Statistical analyses

To analyse differences in stem photosynthesis, hydraulic conductivity, bark total chlorophyll concentration, and NSC concentrations among light exclusion treatments, we used a linear mixed model with fixed factors for light exclusion (control and painted) and cultivar, and given random effects to time (*Before*, *During*, and *Post*) and block (4; well-watered plants).


Modelused:y∼Lighttreatment×Cultivar+(1|Block)+(1|Time)

To analyse differences in NSC concentrations, stem photosynthesis, hydraulic conductivity, soil moisture and midday stem water potential among trees subject to both water and light exclusion *During* the experiment, we used linear mixed models with fixed factors for light treatment (control and painted) and drought treatment (control and droughted) and given random effect for block. We did not use a traditional repeated-measures approach because of the destructive nature of the sampling; the observations were not made on the same branch/leaf over time.


Modelused:y∼Watertreatment×Lighttreatment+(1|Block)

Finally, to analyse the relationship between two traits, such as *A*_stem_ and NSC in the bark, data from each cultivar was separated and analysed separately with a fixed effect of light treatment and given random effects of block and time.


$$e.g.\;(\mathrm{NS}C_{\mathrm{bark}}∼A_{\mathrm{stem}}+\mathrm{Light}\;\mathrm{treatment}+(1|\mathrm{Block})+(1|\mathrm{Time})$$

All analyses were carried out using the package lmerTest in R v4.5.1 (R Core Team 2024).

## Results

### Light exclusion effect on stem photosynthesis

**Figure 2.**
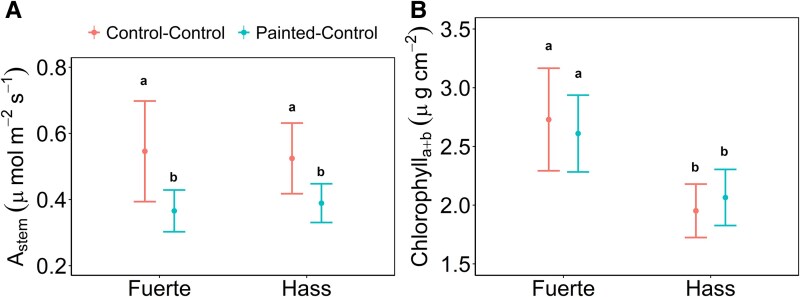
Avocado (a) stem photosynthetic rate (*A*_stem_) and (b) Chlorophyll_a+b_ concentration for the cultivars ‘Fuerte’ and ‘Hass’ under light exclusion treatments (painted and unpainted control) in well-watered conditions (*Before*, *During*, and *Post*). Means (*n* = 12) ± SE.

Light exclusion treatment significantly reduced stem photosynthesis in both avocado cultivars (*P* = .043, *f*_(1,37)_ = 4.41). Stem photosynthesis in painted trees ranged from 0.04 to 0.77 μmol m^−2^ s^−1^ while stem photosynthesis in unpainted trees ranged from 0.02 to 1.55 μmol m^−2^ s^−1^ (Fig. 2a), without statistical differences between cultivars (*P* = .88, *f*_(1,37)_ = 0.02). On the other hand, Chlorophyll_a+b_ in the bark showed no effect of light treatment (*P* = .99, *f*_(1,42)_ = 0.0002), but it was significantly different between cultivars (*P* < .01, *f*_(1,42)_ = 7.6). ‘Fuerte’ demonstrated mean Chlorophyll_a+b_ values of 2.67 ± 0.27 μg cm^−2^, while ‘Hass’ had values of 2.01 ± 0.16 μg cm^−2^ (Fig. 2b).

### Light exclusion effect on stem photosynthesis and non-structural carbohydrates relationships

**Figure 3.**
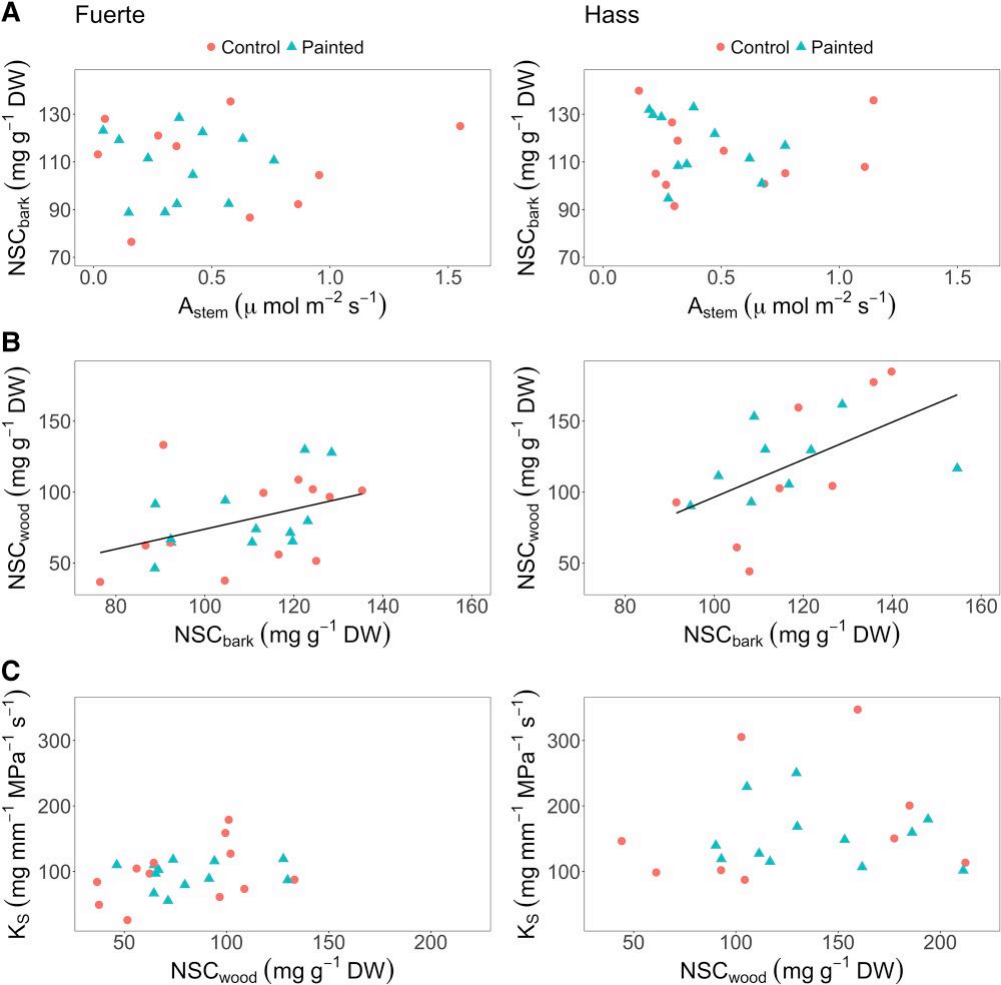
Relationships between (a) stem photosynthetic rate (*A*_stem_) and (NSC: sugars + starch) concentration in the bark tissue, (b) NSC concentration in the bark tissue and NSC concentration in the wood tissue, and (c) NSC concentration in the wood and sapwood-specific hydraulic conductivity (*K*_S_) in avocado stems of cultivars ‘Fuerte’ and ‘Hass’ under light exclusion treatments (*Before*, *During*, and *Post*). Regression lines in (b) come from linear mixed models where no differences between control and painted trees were found. Each point represents an individual tree.

We did not find a significant relationship between stem photosynthesis and (NSC: sugars + starch) concentration in the bark tissue for either ‘Hass’ or ‘Fuerte’ cultivars (‘Hass’: *P* = .64, *f*_(1,4)_ = 0.25; ‘Fuerte’: *P* = .27, *f*_(1,18)_ = 1.29; Fig. 3a). We also found no effect of light exclusion on the concentration of NSCs in the bark of either cultivar (‘Hass’: *P* = .45, *f*_(1,20)_ = 0.60; ‘Fuerte’: *P* = .93, *f*_(1,16)_ = 0.007). However, we observed a significant positive relationship between the concentration of NSCs in the bark and the concentration of NSCs in the wood tissue for both cultivars (‘Hass’: *P* = .029, *f*_(1,15)_ = 5.8; ‘Fuerte’: *P* = .03, *f*_(1,21)_ = 5.4; Fig. 3b). There was no evidence for an effect of the light exclusion treatment on the concentration of NSCs in the wood of either cultivar (‘Hass’: *P* = .53, *f*_(1,15)_ = 0.41; ‘Fuerte’: *P* = .78, *f*_(1,18)_ = 0.08). Finally, we observed no significant relationship between the concentration of NSCs in the wood and sapwood-specific hydraulic conductivity (*K*_S_), (‘Hass’: *P* = .68, *f*_(1,18)_ = 0.17; ‘Fuerte’: *P* = .15, *f*_(1,21)_ = 2.25; Fig. 3c). There was also no significant effect of stem light exclusion on *K*_S_ of either cultivar (‘Hass’: *P* = .53, *f*_(1,18)_ = 0.40; ‘Fuerte’: *P* = .90, *f*_(1,21)_ = 0.01).

### Light exclusion and water stress effects on stem photosynthesis

**Figure 4.**
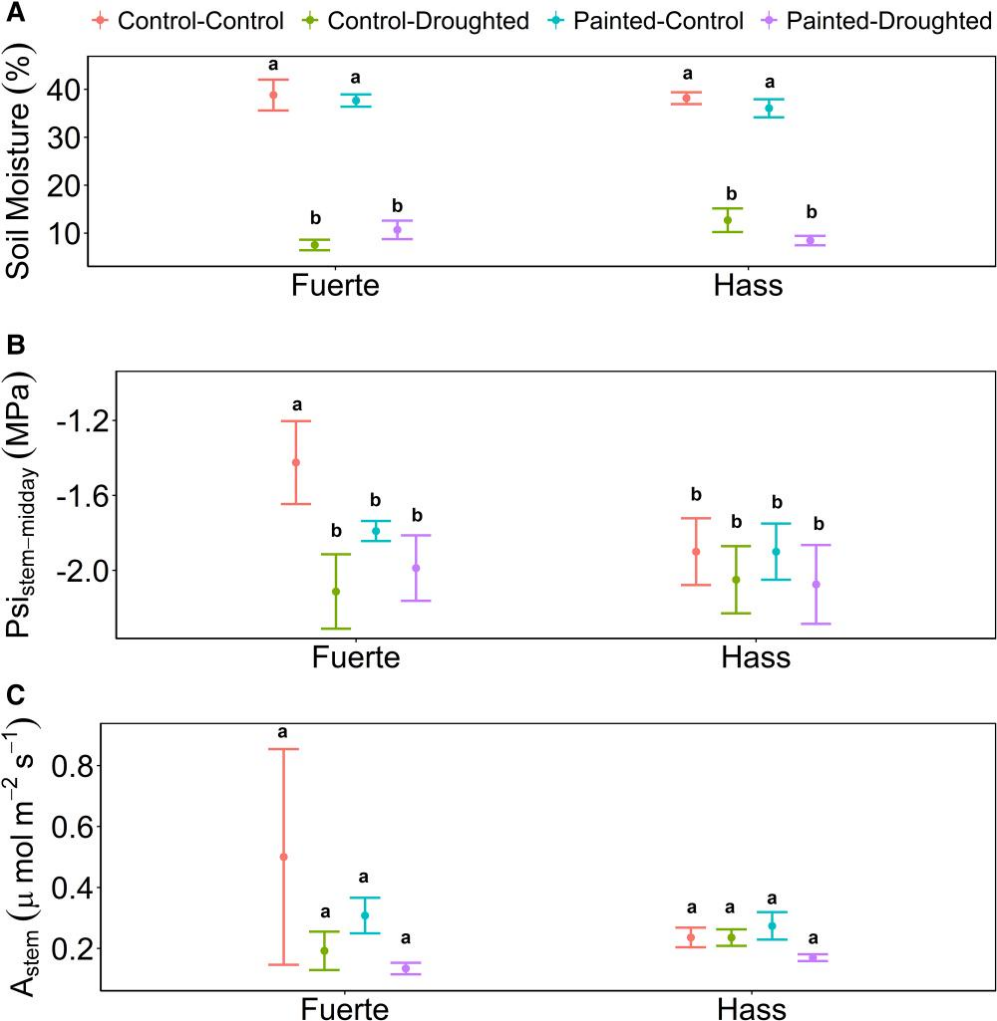
Avocado (a) soil moisture; (b) midday stem water potential (ψ_stem-midday_); and (c) stem phtosynthetic rate (*A*_stem_) for ‘Fuerte’ and ‘Hass’ cultivars under light exclusion and water stress treatments (*During*). Control-control: non-painted and non-water-stressed trees, control-droughted: non-painted and water-stressed trees, painted-control: painted and non-water-stressed trees, and painted-droughted: painted and water-stressed trees. Means (*n* = 4) ± SE.

To test for the interactive effect of light exclusion and water stress, we used the data collected *During* the water stress experiment. Soil moisture demonstrated a significant difference between water stress treatments for both cultivars (*P* < .0001, *f*_(1,28)_ = 412) reaching <15% for stressed trees, while control trees soil moisture was >40%. In addition, there were no light exclusion treatment effects on soil moisture (Fig. 4a).

We did not find a significant effect of light treatment on ψ_stem-midday_ in any of the cultivars (‘Fuerte’: *P* = .48, *f*_(1,9)_ = 0.75; ‘Hass’: *P* = .95, *f*_(1,12)_ = 0.005) (Fig. 4b). However, we found a significant effect of water stress on ψ_stem-midday_ only for non-painted ‘Fuerte’ trees (*P* = .02, *f*_(1,28)_ = 5.94) (Fig. 4b). Cultivars showed different ranges of values of ψ_stem-midday_, with ‘Fuerte’ having values from −1.8 to −1.2 MPa for control trees and from −2.3 to −1.8 MPa for water-stressed trees. On the other hand, ‘Hass’ trees had values from −2 to −1.8 MPa for control trees and from −2.3 to −1.9 MPa for water-stressed trees (Fig. 4b).

Finally, stem photosynthesis did not differ among water stress or light exclusion treatments in either of the two cultivars (cultivars: *P* = .56, *f*_(1,24)_ = 0.35; light treat: *P* = .46, *f*_(1,24)_ = 0.56; water stress treat: *P* = .13, *f*_(1,24)_ = 2.51; Fig. 4c).

### Light exclusion and water stress effects on stem photosynthesis and non-structural carbohydrates relationships

**Figure 5.**
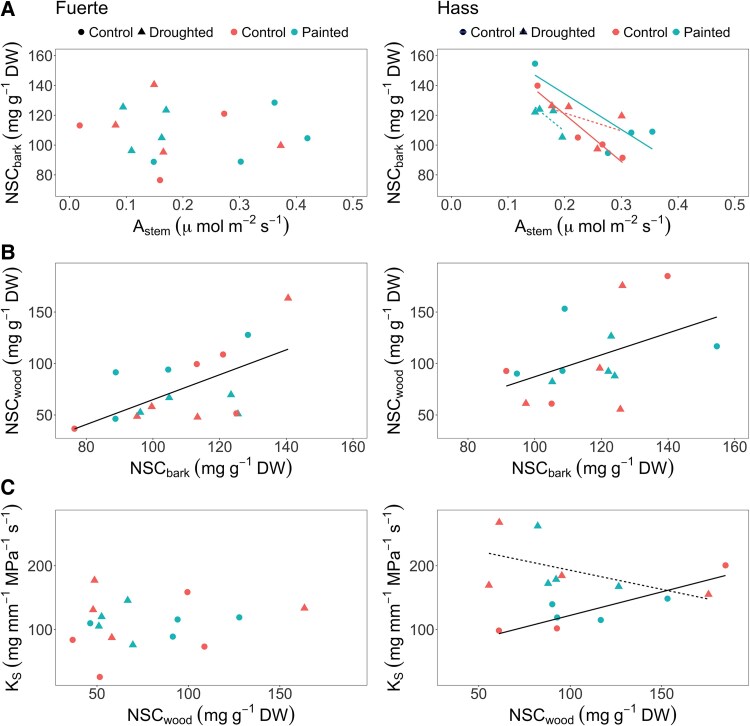
Relationship between (a) stem photosynthetic rate (*A*_stem_) and (NSC: sugars + starch) concentration in the bark tissue for ‘Fuerte’ and ‘Hass’ avocado plants under light exclusion and water stress treatments (*During*). For ‘Hass’ (left panel), lines are fitted to significant relationships for light and water treatments, solid line for control and dashed line for droughted, and colors indicate painted and non-painted trees. (b) Relationship between NSC concentration in the bark and wood tissues, black solid lines are fitted to the significant relationship between the variables, while there were no treatment effects. (c) Relationship between NSC concentration in the wood and sapwood-specific hydraulic conductivity (*K*_S_). For ‘Hass’, lines are fitted to water treatments, solid line for control and dashed line for drought. Each point represents an individual tree.

For trees subject to both water stress and light exclusion treatments, there was a significant relationship between stem photosynthesis and NSC concentration in the bark tissue for the ‘Hass’ cultivar only (‘Hass’: *P* < .001, *f*_(1,11)_ = 20.01; ‘Fuerte’: *P* = .34, *f*_(1,11)_ = 0.98). Additionally, ‘Hass’ trees demonstrated a significant interaction between water stress and light exclusion treatments (‘Hass’: *P* = .035, *f*_(1,11)_ = 5.78; ‘Fuerte’: *P* = .82, *f*_(1,11)_ = 0.05), where painted trees had a similar negative relationship for both water-stressed and control trees, while non-painted trees had a stronger negative relationship between NSCs in the bark and *A*_stem_ in control trees compared with stressed trees (Fig. 5a).

For the relationship between NSC concentration in the bark and wood tissues, there was a significant relationship in both cultivars (‘Hass’: *P* = .0052, *f*_(1,7)_ = 15.25; ‘Fuerte’: *P* = .0061, *f*_(1,11)_ = 11.45), although there were no water stress or light exclusion treatment effects (Fig. 5b).

With respect to the relationship between NSC concentration in wood tissue and *K*_S_, there was a significant effect of water stress only for the ‘Hass’ cultivar (‘Hass’: *P* = .0064, *f*_(1,7)_ = 14.12; ‘Fuerte’: *P* = .19, *f*_(1,11)_ = 1.99), whereby control trees had a positive relationship between NSC concentration in wood tissue and *K*_S_, while water-stressed trees had a negative one (Fig. 5c).

Interestingly, *K*_S_ was not reduced during water stress, but contrary to what was expected, it increased in both cultivars (Table 2). Moreover, the ratio between starch and sugar concentration in the wood and in the bark was reduced in water-stressed trees by 47% in the wood and 75% in the bark for ‘Fuerte’ and by 40% in both wood and bark for ‘Hass’.

**Table 2.** Mean stem photosynthetic rate (*A*_stem_, μmol m^−2^ s^−1^), sapwood-specific hydraulic conductivity (*K*_S_, mg mm MPa^−1^ s^−1^), sugar and starch concentration, and the ratio of starch to sugar in wood and bark in control (non-painted) plants *Before*, *During*, and *Post* a drought treatment imposed on potted individuals of ‘Hass’ and ‘Fuerte’ avocado trees.

Time	Water treatment	Cultivar	*K*_S_	Wood			Bark		
				Sugar	Starch	Starch:sugar	Sugar	Starch	Starch:sugar
			mg mm MPa^−1^ s^−1^	mg g^−1^	mg g^−1^		mg g^−1^	mg g^−1^	
*Before*	Control	Fuerte	95.26 *b*	48.49 *ns*	17.43 *b*	0.50 *b*	97.71 *ns*	5.14 *b*	0.06 *b*
		Hass	186.16 *a*	49.81 *ns*	66.52 *a*	1.39 *a*	95.13 *ns*	17.47 *a*	0.20 *a*
*During*	Control	Fuerte	96.96 *b b*	43.59 *ns ns*	38.42 *b ns*	0.84 *b ns*	98.67 *ns b*	7.13 *b a*	0.07 *b a*
		Hass	130.95 *a b*	46.40 *ns ns*	66.70 *a ns*	1.58 *a ns*	94.32 *ns b*	18.60 *a a*	0.20 *a a*
	Droughted	Fuerte	122.06 *b a*	47.76 *ns ns*	21.99 *b ns*	0.44 *b ns*	109.99 *ns a*	2.36 *b b*	0.02 *b b*
		Hass	194.67 *a a*	49.35 *ns ns*	47.75 *a ns*	0.92 *a ns*	105.05 *ns a*	12.84 *a b*	0.12 *a b*
*Post*	Control	Fuerte	96.46 *b ns*	58.73 *ns a*	33.99 *b a*	0.61 *b a*	114.24 *ns ns*	4.13 *b a*	0.03 *b a*
		Hass	175.07 *a ns*	53.14 *ns a*	113.38 *a a*	2.19 *a a*	93.19 *ns ns*	31.10 *a a*	0.34 *a a*
	Droughted	Fuerte	81.09 *b ns*	42.57 *ns b*	18.12 *b b*	0.49 *b b*	101.93 *ns ns*	4.03 *b b*	0.04 *b b*
		Hass	166.79 *a ns*	48.70 *ns b*	70.47 *a b*	1.53 *a b*	109.39 *ns ns*	14.46 *a b*	0.14 *a b*

Different letters denote statistical differences *P* < 0.05. First letter shows statistical differences between cultivars, second letter shows statistical differences for water treatment. Light treatment had no statistical differences at any time point (mean, *n* = 8).

## Discussion

We evaluated the effect of stem photosynthesis on hydraulic functioning and drought tolerance and recovery of two avocado tree cultivars. We found that light exclusion, achieved by painting the stems and effectively blocking incident light (Valverdi et al. 2023a), decreased stem photosynthetic re-assimilation rate in both cultivars, but did not affect chlorophyll concentration. We also found that water stress negatively affected midday stem water potential, as expected, but only in non-painted trees of the ‘Fuerte’ cultivar. Interestingly, we identify some relationships between NSCs concentration in the bark and wood during the drought period; but most notably, we observed no effect of stem photosynthesis (*A*_stem_) on NSCs and weak-to-no effects of NSCs on sapwood-specific hydraulic conductivity (*K*_S_). Our results suggest that, in this species, stem photosynthesis is not a main driver for hydraulic function.

First, in contrast to stem net photosynthesis, we found in this study that the stems of avocado carry out stem recycling photosynthesis due to the lack of stomata (data not shown) (Ávila et al. 2014). The rates of stem photosynthesis we measured, 0.2–1 μmol m^−2^ s^−1^, are comparable to rates we have previously observed among 10 different cultivars of avocado using identical methods (Valverdi et al. 2023b). Second, we found a strong effect of light exclusion on the rate of stem photosynthetic re-assimilation in both cultivars, but interestingly there was no effect of light exclusion on bark chlorophyll concentration in either of the two cultivars. Other studies completed in chaparral species showed that plants decreased their stem xylem and phloem chlorophyll concentration during light exclusion (Saveyn et al. 2010) and attributed the observed decrease in stem photosynthesis to these reductions. One reason why we did not see a decrease in bark chlorophyll concentration could be the length of the light exclusion treatment, which was 4 weeks in our experiment, whereas the experiment by Saveyn et al. (2010) was 12 weeks.

We expected that by reducing stem photosynthesis through light exclusion, we would affect the concentration of NSCs in both the bark and wood tissues of stems. However, we found no effect of stem photosynthetic re-assimilation rate on the NSC concentration in either tissue of either cultivar given light exclusion alone. The rates of stem re-assimilation that we measured are towards the low end of the range of values found in the literature for a variety of species (global median of 1.9 μmol m^−2^ s^−1^; Ávila et al. 2014), and this relatively low rate of stem photosynthesis in avocado may not be the main source of the NSC pools found in stems, especially as leaf photosynthesis is around 15 μmol m^−2^ s^−1^ (Acosta-Rangel et al. 2018). These results contrast with those found in other systems. For example, a study in *Populus nigra* showed that shading entire plants with net led to depletion of stem NSC reserves, mainly starch (Tomasella et al. 2021). A depletion in starch after only shading stems was found in *Populus tremula* (De Roo et al. 2020a) and *Fraxinus ornus* (Natale et al. 2023). In these last two species, stem photosynthesis seems to be contributing to starch concentration in stems.

During drought, we expected that those plants with painted stems would suffer stronger declines in their physiological performance, but we only saw a negative effect of light exclusion, drought, or the combination of both in the ‘Fuerte’ cultivar. Individuals of ‘Fuerte’ subjected to light exclusion, drought, or both had lower stem midday water potential and photosynthetic stem re-assimilation. Plants from the ‘Hass’ cultivar already had low values of photosynthetic stem re-assimilation and stem midday water potential in the control-control group despite having high soil moisture and did not respond to any of the treatments. There is evidence that ‘Hass’ may have a stronger response to drought than ‘Fuerte’, both with respect to plant water potentials and the associated effects on photosynthesis and drought (Chartzoulakis et al. 2002). In addition, we note that trees were too big at the end of the growing season for the 20 l pots, which could cause water deficit due to the high LA to soil volume ratio restricting root development and causing more negative stem water potential values even in well-watered trees.

We also expected that plants subject to light exclusion would have reduced *K*_S_ during drought. However, our results showed no effect of light treatment on hydraulic conductivity *Before*, *During*, or *Post* drought, meaning that, under the conditions evaluated, there is no clear effect of stem photosynthesis on the hydraulic functioning of avocado trees (Table 1). Our results are consistent with a previous observational study among 10 different cultivars of avocado, whereby we observed almost no correlation between hydraulic conductivity and sugar or starch concentrations in the bark or wood (Valverdi et al. 2023b). Nevertheless, the maintenance of hydraulic functioning during drought may be linked to a re-mobilization of NSCs from starch to sugar in the wood of the stems in both ‘Fuerte’ and ‘Hass’ plants. These results are also consistent with our previous observational study, which found a significant positive correlation between the ratio of starch to sugar in wood and hydraulic conductivity. The conversion of starch to soluble sugars in the wood would draw in water that can be used for maintaining hydraulic conductivity and/or for xylem refilling after loss of conductivity. This osmotic adjustment has been observed in other species with stems that have notable rates of photosynthesis (Nardini et al. 2011, Secchi and Zwieniecki 2016, Tomasella et al. 2021) and in dehydration-tolerant species as mentioned by Pratt et al. (2021).

Overall, our results suggest that the role of stem photosynthesis on drought performance is not particularly important in avocado trees, especially given that it does not affect hydraulic functioning or recovery after drought. This does not mean that stem photosynthesis could not be important for processes/scales not considered in this study, such as for short-distance transport of photoassimilates or in events when leaves do not contribute to carbon gain (i.e. a specific phenological stage or when they have fallen due to abiotic/biotic stress). Comparing the two cultivars, it appears that ‘Fuerte’ is more drought tolerant than ‘Hass’ (Chartzoulakis et al. 2002), which suggests that more efforts should be directed to increase the viable cultivation of ‘Fuerte’ in Southern California. New rootstock and scion cultivars of avocado could also be surveyed and bred to be capable of performing higher stem photosynthesis, which could potentially respond better to a warmer and drier environment, especially in the Mediterranean region of southern California.

## Supplementary Material

plaf044_Supplementary_Data

## Data Availability

Data and code used for statistical analysis in this study are available on Zenodo (Goldsmith et al. 2025).
