# Supplementary material for: Does green stem photosynthesis affect plant drought tolerance and recovery in avocado?
Source: AoB Plants. 2025 Aug 23;17(5):plaf044. doi: 10.1093/aobpla/plaf044 (PMC12448719; doi:10.1093/aobpla/plaf044)
Supplement: plaf044_Supplementary_Data [file plaf044_supplementary_data.pdf]

Supporting Information: Does green stem photosynthesis affect plant drought tolerance and recovery in avocado?

Valverdi et al.

BLOCK 1		BLOCK 2		BLOCK 3		BLOCK 4	
H N C	H E D	H E D	F N D	F N D	F E C	F N D	H E D
F E C	H E C	H E C	F E C	F E D	F N C	F N D	H N C
H E C	H E D	H N D	F N D	H E C	H E C	H N D	H E C
H N C	F E D	H E C	F E C	H E D	F E D	F E C	F E C
F E D	H N D	H E D	F N C	F E D	F E D	F E D	F E D
F N D	F N C	H N D	F E D	H E D	F E C	H E C	F N C
F N D	F E C	F E D	F N C	H E D	F E C	H E C	H E D
H E D	F E C	F E C	H N C	H N C	H E C	H E D	H E D
F E C	H E D	H N C	F E D	F E C	F N D	H E C	H N C
H N D	H E D	H E D	F E C	H N C	F N C	H E D	F E C
F E D	H E C	H E C	H E D	H N D	H E D	F E D	F E D
H E C	F N C	F E D	H E C	H N D	H E C	F E C	F N C

Avocado cultivars	Light treatments	Water stress treatments
H: Hass (white)	E: Light exclusion	D: Drought
F: Fuerte (grey)	N: No light exclusion	C: Control

Figure S1. Complete randomized block experimental design with three factors: avocado cultivars, light exclusion treatments and water stress treatments, where each factor has two levels. Twelve trees per cultivar in each block were randomly assigned to the light and water stress treatments, giving a total of 24 trees per block (four trees per combination cultivar x light treatment x water stress treatment) yielding a total of 96 trees for the whole experiment.

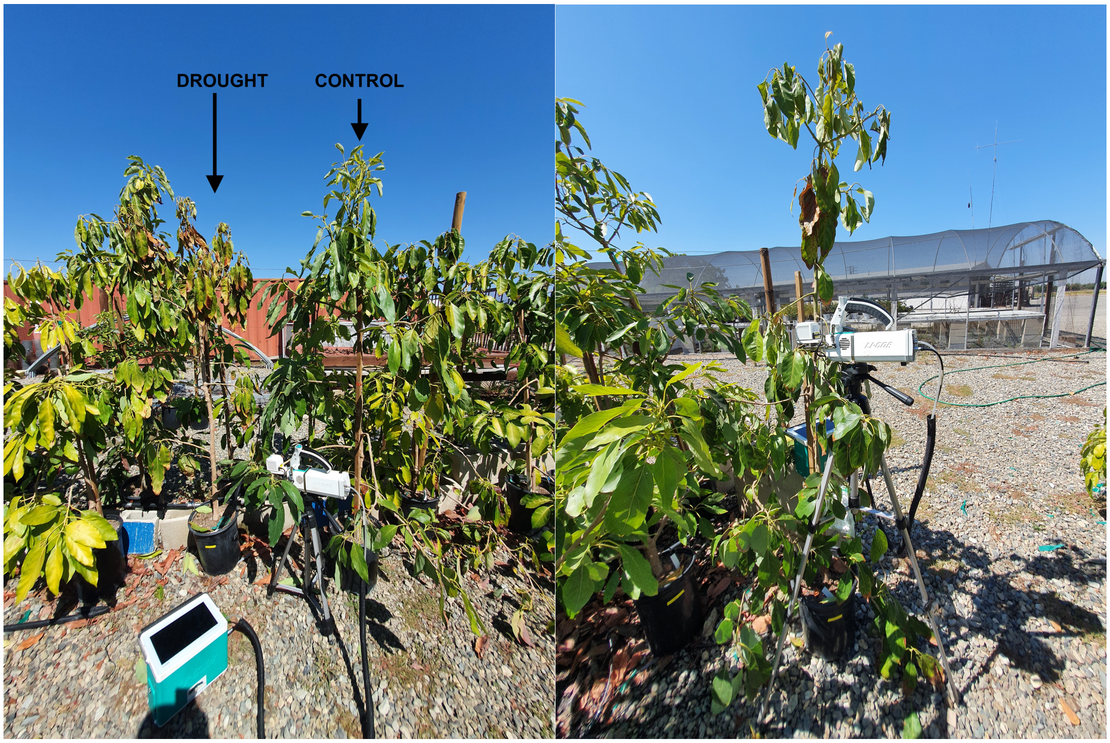

Figure. S2. Avocado trees (left) at the conclusion of the drought experiment and (right) under water stress at wilting point and showing signs of leaf necrosis.
